# Genome-Wide Identification and Functional Analysis of *AP2/ERF* Gene Family in *Passiflora edulis* Sims

**DOI:** 10.3390/plants14050645

**Published:** 2025-02-20

**Authors:** Lanjun Luo, Liping Zhang, Ronghao Gu, Shihao Ni, Jingyao Yu, Yachao Gao, Chuanying Fang

**Affiliations:** 1 School of Breeding and Multiplication (Sanya Institute of Breeding and Multiplication), Hainan University, Sanya 572025, China; 2 School of Tropical Agriculture and Forestry, Hainan University, Danzhou 571737, China; 3 School of Life and Health Sciences, Hainan University, Haikou 570288, China; 4 Baoting Research Institute, Hainan University, Baoting 572300, China

**Keywords:** passion fruit, AP2/ERF, postharvest storage, *PeSTP6*, flavor

## Abstract

The Apetala2/Ethylene Responsive Factor (AP2/ERF) family represents a critical group of transcription factors in plants, recognized for their roles in growth, development, fruit ripening, and postharvest processes. This study aimed to identify and characterize the *AP2/ERF* gene family in passion fruit (*Passiflora edulis* Sims) and investigate their potential roles in flavor enhancement. A total of 91 *PeAP2/ERF* genes were identified and classified into five subfamilies. Chromosome localization and collinearity analysis demonstrated their distribution across all nine chromosomes of passion fruit, with tandem duplication events identified as a key driver of family expansion. Exon–intron configurations and motif compositions were highly conserved among *PeAP2/ERF* genes. Promoter cis-acting element analysis indicated potential regulation by environmental signals, including abiotic and biotic stresses, as well as hormonal cues. Postharvest storage induced the expression of 59 *PeAP2/ERF* genes over time. Notably, *PeAP2-10* was found to enhance the expression of *PeSTP6*, a gene associated with sugar transport, suggesting its potential influence on the flavor profile of passion fruit. These findings provide valuable insights into the functional roles of *PeAP2*/*ERF* genes in passion fruit, highlighting their significance in postharvest management and flavor quality enhancement strategies.

## 1. Introduction

Among the transcription factor families, the Apetala2/Ethylene Responsive Factor (AP2/ERF) family is recognized as one of the most conserved and plant-specific families, playing a crucial role in various plant species [1]. Members of the AP2/ERF family possess at least one highly conserved AP2 domain, which consists of 60 to 70 amino acids. Based on the number of AP2 domains and other DNA-binding domains, the AP2/ERF superfamily can be divided into five subfamilies: APETALA2 (AP2), dehydration responsive element binding (DREB), ethylene responsive element binding protein (ERF), related to ABI3/VP (RAV), and Soloist. The AP2 subfamily contains two AP2 domains, and the other subfamilies have a single AP2 domain [2,3].

*AP2/ERF* family genes are essential for plant development [4]. For instance, in Arabidopsis, *AtERF-13* regulates lateral root growth and development by modulating long-chain fatty acid synthesis [5]; in poplar, *ERF139* promotes xylem expansion following its expression in the stems [6]; in *Liriodendron chinensis*, *AP2/ERF* family genes are involved in the regulation of leaf growth and development [7]; in soybean, *TOE4b* regulates photoperiodic flowering [8]; in sweet cherry, *PavRAV2* negatively regulates fruit size by directly inhibiting *PavKLUH* expression [9]; in *Perilla* seeds, three AP2/ERFs (WRI, ABI4, and RAVI) regulate oil production [10]. In addition to their roles in growth and development, *AP2/ERF* genes also respond to environmental stimuli and hormonal signals. In *Brassica napus* leaves, 118 *AP2/ERF* family genes are likely to enhance cold tolerance through various molecular pathways [11]. In *Eschscholzia California* (California poppy), *AP2/ERF* genes respond to MeJA, with 20 family members being up-regulated [12]. Recent studies have indicated that *AP2/ERFs* also play significant roles in fruit development and postharvest. For example, *FcAP2*/*ERF* regulates the rapid ripening of fig fruit [13]. In addition, *PgAP2/ERF5*, *PgAP2*/*ERF36*, *PgAP2*/*ERF58*, and *PgAP2*/*ERF86* are associated with fruit hardness, and the *PgAP2/ERF* gene family is a potentially important regulator of pomegranate fruit development. It has been shown that *AP2/ERFs* are abundantly expressed in postharvest fruits and respond to environmental and hormonal responses. Notably, more than half of the *PgAP2*/*ERFs* are expressed at relatively lower levels in postharvest pomegranate fruits under low temperature storage. Furthermore, *PgAP2*/*ERF4*, *PgAP2*/*ERF15*, *PgAP2*/*ERF26*, *PgAP2*/*ERF30*, *PgAP2*/*ERF35*, and *PgAP2*/*ERF45* genes are up-regulated in treatments that promote pomegranate postharvest preservation [14]. Studies on the function and regulatory mechanisms of *AP2/ERF* in fruit ripening and postharvest storage have found that CpERF9 specifically binds to the promoters of *CpPME1/2* and *CpPG5* in papaya fruits, represses promoter activity, and regulates fruit ripening [15]. In loquat, EjAP2-1 binds to *EjMYB1/2* and depresses *Ej4CL1* promoter activity, consequently regulating phenylpropanoid metabolism under cold stress [16]. However, the role of *AP2/ERF* genes in the postharvest storage of passion fruit remains unclear.

Passion fruit (*Passiflora edulis* Sims), a tropical and subtropical crop with year-round fruiting and high nutritional value, has recently gained attention for genomic and flavor metabolism studies. In recent years, biological research on passion fruit has increasingly focused on its genome and flavor metabolism. Among these, 174 MYB family members and 14 SBP family members have been identified, and both are primarily involved in abiotic stresses, such as temperature, drought, and salt stress [17,18]. The promoter regions of *PebHLHs* are involved in developmental regulation, hormonal responses, and stress responses. In the context of flavor metabolism in passion fruit, it has been discovered that the induced *PeLOX4* expression results in an increase in LOX enzyme activity, which in turn, promotes the synthesis of volatile esters in the pulp and enhances the aroma of the fruit [19]. Transient overexpression of *PeCWINV5* in passion fruit increases soluble sugar accumulation [20]. Multiomics analyses of passion fruit have revealed that the *ACX*, *ADH*, *ALDH*, and *HPL* gene families are key regulators for ester synthesis, especially *ACX13/14/15/20*, *ADH13/26/33*, *ALDH1/4/21*, and *HPL4/6*. The Terpene Synthase (TPS) gene family is crucial for terpenoid synthesis, especially *PeTPS2/3/4/24* [21]. Recently, our work has indicated that low temperatures and MeJA effectively preserve the fruit and delay the aging process of passion fruit during postharvest storage [22]. Nevertheless, the role of *AP2*/*ERFs* in the postharvest preservation of passion fruit remains unknown.

Considering that *AP2/ERF* genes are responsive to hormones and low temperature, and given their biological functions in plant growth, especially in the postharvest senescence of fruit [23], we hypothesized that this gene family may play crucial roles in the postharvest preservation of passion fruit. In this study, we carried out a genome-wide characterization of *AP2/ERF* genes and investigated the regulation of downstream genes in postharvest passion fruit. Our results provide key information regarding the *AP2*/*ERF* genes of passion fruit and present important experimental data on the role of *PeAP2*/*ERF* genes in the postharvest preservation of passion fruit.

## 2. Results

### 2.1. Identification of Ninety-One PeAP2/ERF Genes in the Passion Fruit Genome

A BLASTp analysis utilizing AtAP2/ERF sequences as query sequences led to the identification of 91 AP2/ERF-encoding genes within the passion fruit genome. After removing redundant sequences and alternative transcripts corresponding to the same genes, the *PeAP2/ERF* genes were designated according to their order in the passion fruit genomic sequence database (Table 1). The lengths of PeAP2/ERF proteins ranged from 118 amino acids (PeERF-47) to 1209 amino acids (PeDREB-2), and their molecular weights (MW) ranged from 7.22 kDa (PeERF-47) to 13.31 kDa (PeDREB-2). The predicted isoelectric points (*p*I) of the PeAP2/ERF proteins ranged from 4.26 (PeDREB-19) to 10.87 (PeERF-47). Notably, 52 proteins exhibited *p*Is ≥ 7, indicating a positive charge in acidic solutions. The hydrophilic indices of all proteins ranged from −1.267 to 0.749, reflecting their varying degrees of hydrophobicity. In summary, the 91 *AP2/ERF* genes in passion fruit showed significant variations in their physical and chemical properties (Table 1).

### 2.2. PeAP2/ERF Proteins Are Classified into Five Subfamilies

**Figure 1:**
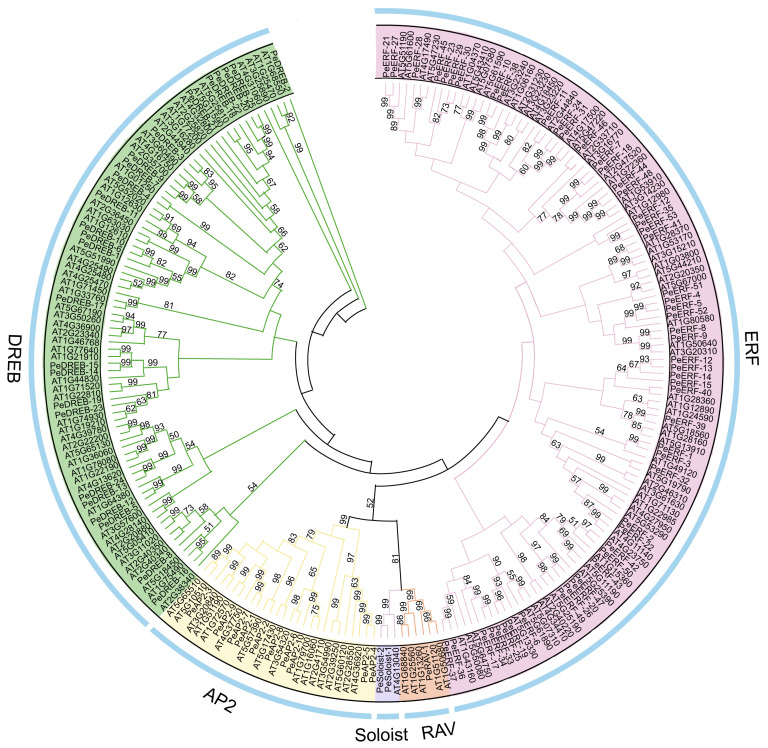
Phylogenetic relationships of AP2/ERF proteins from Arabidopsis and passion fruit. A total of 141 AtAP2/ERFs proteins from Arabidopsis and 91 PeAP2/ERFs from passion fruit were utilized to construct a phylogenetic tree using the Neighbor-Joining (NJ) method implemented in MEGA12 software. The numbers on the branches represent bootstrap values calculated from 1000 replicates. All AP2/ERF proteins were classified into five subfamilies: AP2, DREB, ERF, RAV, and Soloist, which are represented by yellow, green, pink, tawny, and purple, respectively.

To investigate the evolutionary relationships among the passion fruit PeAP2/ERF family members, a phylogenetic tree was constructed by aligning the full-length protein sequences of 141 *A. thaliana* and 91 passion fruit sequences (Figure 1). The AP2/ERF family members were grouped into five subfamilies. The ERF subfamily comprised 53 members, the DREB subfamily contained 25 members, and the AP2 subfamily consisted of 10 members, together accounting for approximately 96.7% of the PeAP2/ERF family members; the Soloist and RAV subfamilies contained 2 and 1 member, respectively (Figure 1). Notably, the similarity among members of the same subfamily was higher in both passion fruit and *A. thaliana*. This accurate classification of AP2/ERF protein family members, based on their shared structural characteristics, highlights their high similarity across different species.

### 2.3. Chromosome Localization and Collinearity Analysis of the PeAP2/ERF Genes

**Figure 2:**
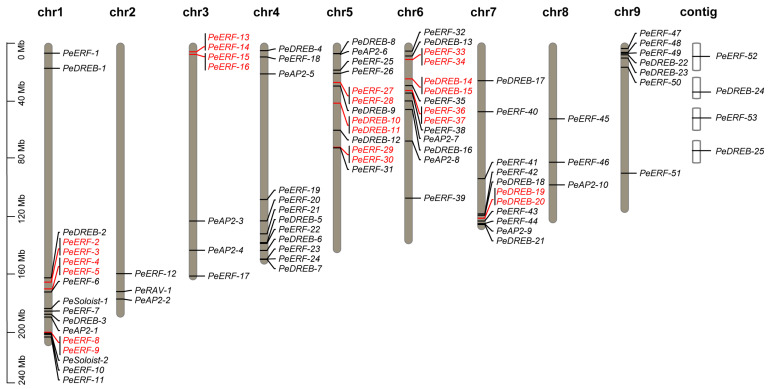
Chromosomal distribution and tandem duplication analysis of *PeAP2/ERF* genes. Each bar represents a chromosome, and tandemly duplicated gene pairs are highlighted in red. The approximate locations of each *PeAP2/ERF* gene on each chromosome are indicated.

The 91 *PeAP2/ERF* genes were distributed across all nine chromosomes of the passion fruit. Specifically, *PeERF-1* to *PeERF-11*, *PeDREB-1* to *PeDREB-3*, *PeSoloist-1* to *PeSoloist-2*, and *PeAP2-1* were located on chromosome 1; *PeERF-12*, *PeRAV-1*, and *PeAP2-2* were located on chromosome 2; *PeERF-13* to *PeERF-17*, and *PeAP2-3* to *PeAP2-4* were located on chromosome 3; *PeERF-18* to *PeERF-24*, *PeDREB-4 to PeDREB-7*, and *PeAP2-5* were located on chromosome 4; *PeERF-25* to *PeERF-31*, *PeDREB-8* to *PeDREB-12*, and *PeAP2-6* were located on chromosome 5; *PeERF-32* to *PeERF-39*, *PeDREB-13* to *PeDREB-16*, and *PeAP2-7* to *PeAP2-8* were located on chromosome 6; *PeERF-40* to *PeERF-44*, *PeDREB-17* to *PeDREB-21*, and *PeAP2-9* were located on chromosome 7; *PeERF-45* to *PeERF-46* and *PeAP2-10* were located on chromosome 8; *PeERF-47* to *PeERF-51* and *PeDREB-22* to *PeDREB-23* were located on chromosome 9. Additionally, *PeERF-52* to *PeERF-53* and *PeDREB-24* to *PeDREB-25* were located on unanchored scaffolds (Figure 2).

Further collinear analysis using MCScanX (v1.0.0) revealed that many gene members had collinearity within or between passion fruit chromosomes. The results indicated the presence of 12 tandem duplication gene pairs: *PeERF-2* and *PeERF-3*, *PeERF-4* and *PeERF-5*, *PeERF-8* and *PeERF-9*, *PeERF-13* and *PeERF-14, PeERF-15* and *PeERF-16, PeERF-27* and *PeERF-28*, *PeERF-29* and *PeERF-30*, *PeERF-33* and *PeERF-34*, *PeERF-36* and *PeERF-37*, *PeDREB-10* and *PeDREB-11*, *PeDREB-14* and *PeDREB-15,* and *PeDREB-19* and *PeDREB-20*. These tandem duplication events significantly contribute to the diversity and evolutionary history of gene families and play a crucial role in understanding the adaptive evolution of species (Figure 2).

**Figure 3:**
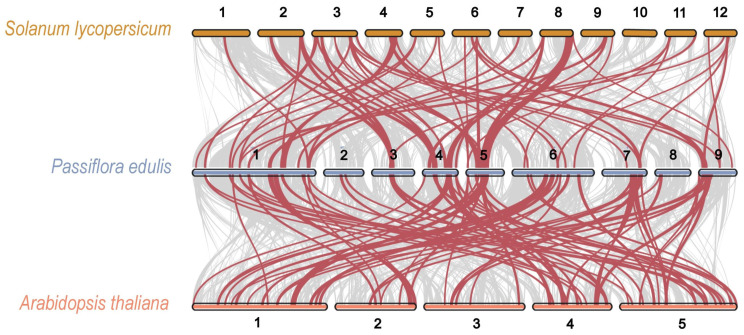
Collinearity analysis of *PeAP2/ERF* genes between passion fruit and two other species (*A. thaliana* and *Solanum lycopersicum*). The grey lines in the background represent collinear blocks between passion fruit and other plant genomes, while the wine-red lines highlight the collinear *PeAP2/ERF* gene pairs. The chromosomes of Arabidopsis, passion fruit, and tomato are represented by tangerine, blue-grey, and tan bars, respectively, with numbers representing the respective chromosomes.

Furthermore, a multicollinearity analysis was carried out to identify robust orthologs of passion fruit *AP2*/*ERF* genes in the genomes of other species, specifically *A. thaliana* (Figure 3). The analysis revealed that the collinear gene pairs among these species had undergone lineage-specific expansions during evolution. The results showed that the highest collinearity was observed between passion fruit and tomato, followed by passion fruit and Arabidopsis. Chromosome 1 exhibited the highest number of orthologs with all other species. Overall, the maximum number of collinear orthologs was found between passion fruit and Arabidopsis, suggesting that the *AP2*/*ERF* genes were conserved and likely shared common ancestors, with the exception of instances of duplication or loss (Figure 3).

### 2.4. The Structure of PeAP2/ERFs Is Highly Conserved

**Figure 4:**
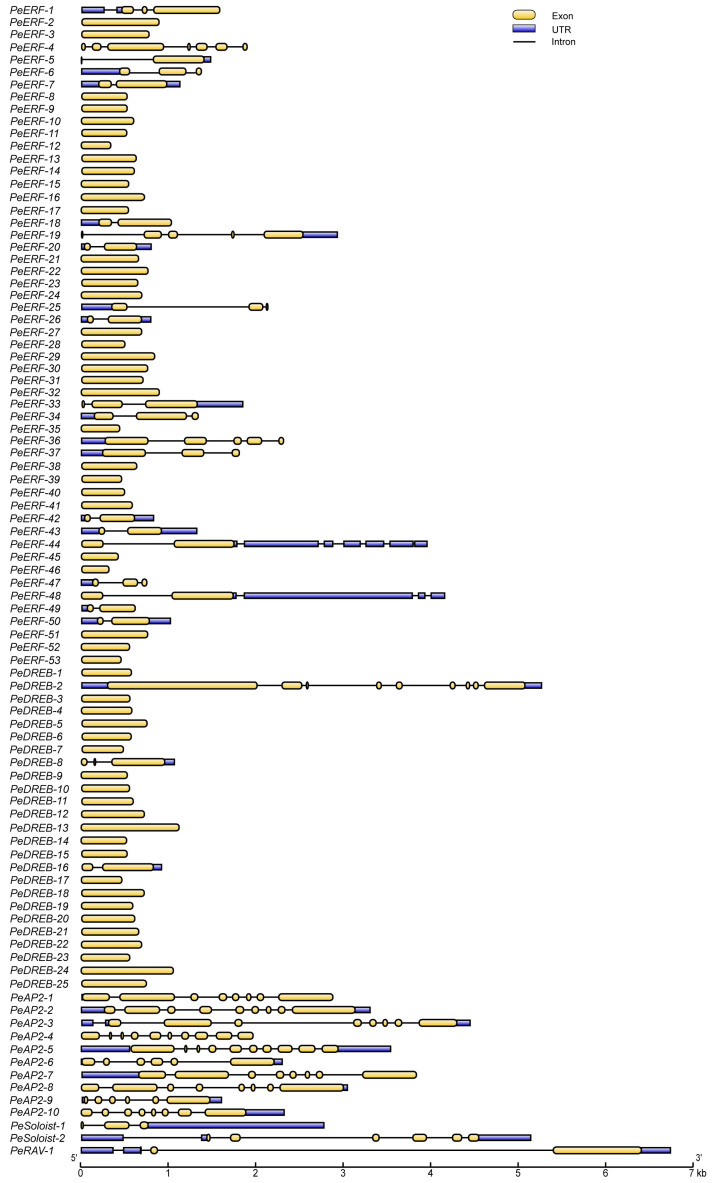
Structure of *PeAP2/ERF* genes. Exons and introns are represented with yellow boxes and black lines, respectively. Blue boxes refer to the untranslated regions (UTRs). The exon and intron lengths are calculated according to the scale at the bottom.

We analyzed the structure of the *PeAP2/ERF* genes to gain further insights into their functional and evolutionary diversification. The intron–exon structures were plotted according to subfamily order in the phylogenetic tree (Figure 4). Based on sequence similarity, all *PeAP2/ERF* genes comprised 5′- or 3′-noncoding regions (UTRs), 1 to 10 exons, and zero to nine introns (Figure 4). Specifically, *PeAP2-4* and *PeAP2-5* in the subfamily *AP2* contained 10 exons. Other *PeAP2* members had six to nine exons. *PeERF* members had one to seven exons. In the *PeERF* subfamily, 28 genes contained one exon, and none of these members had UTRs. *PeDREB* genes typically lacked introns and UTRs, except for *PeDREB-2*, *PeDREB-8*, and *PeDREB-16*, which had UTRs and at least two exons. *PeRAV-1* contained three exons. *PeSoloist-1* and *PeSoloist-2* contained three and six exons, respectively (Figure 4). Additionally, *PeAP2/ERFs* in the duplication pairs exhibited similar exon and intron lengths and numbers (Figure 4).

**Figure 5:**
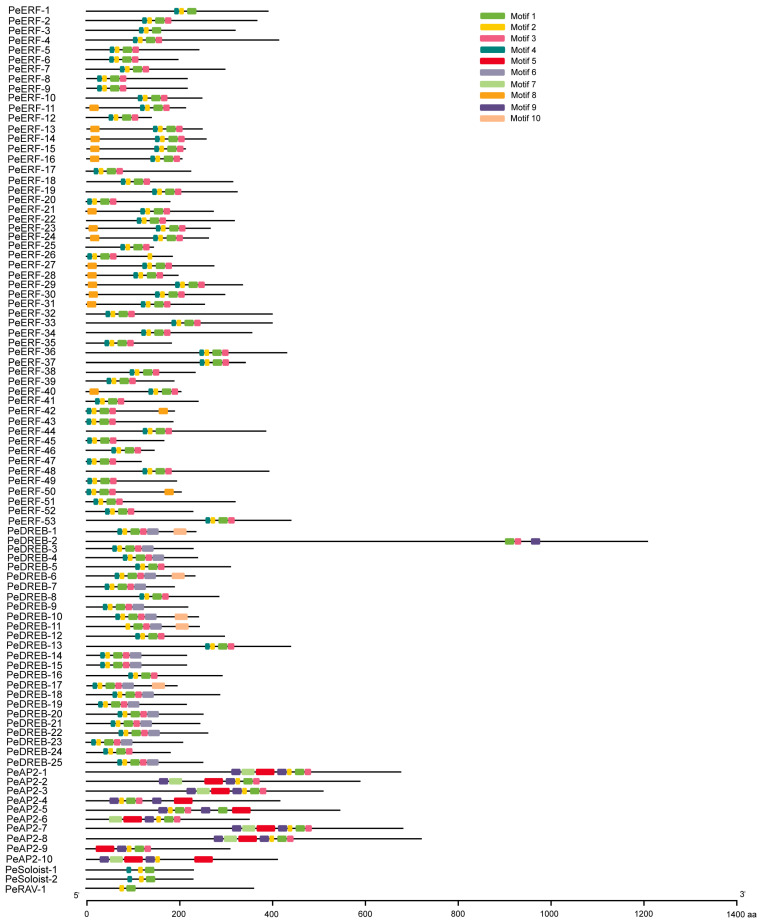
The conserved motifs of PeAP2/ERF proteins. Motifs are shown in the order in which they are arranged in the original phylogenetic tree. Each motif is represented by a colored box and the nonconserved sequences are presented with black lines. The length of the motif is indicated by the width of the image (unit: amino acids).

Using TBtools software (v2.121), we identified 10 motifs in PeAP2/ERF proteins, with lengths ranging from 8 to 41 amino acids (Figure 5 and Figure S1). Specifically, the PeAP2 subfamily contained seven motifs, including motifs 1, 2, 3, 5, 7, 9, and 10. Among them, the subfamily members shared the conserved motifs of 2, 5, and 9. The PeERF subfamily contained motifs 1, 2, 3, 4, and 8. The PeDREB subfamily contains motifs 1, 2, 3, 4, 6, 7, and 10. Among these, the PeERF and PeDREB subfamilies shared the conserved motifs of motifs 1 and 2; however, the conserved motif unique to the PeERF subfamily was motif 4, while the conserved motif unique to the PeDREB subfamily was motif 3. The PeSoloist subfamily contained motifs 1, 2, 3, and 4. The PeRAV subfamily contained motifs 1 and 2. Both the PeSoloist and PeRAV subfamilies had only one conserved motif (motif 1), which may serve as AP2 domain (Figure 5).

### 2.5. Identification of Cis-Acting Elements in PeAP2/ERF Promoters

**Figure 6:**
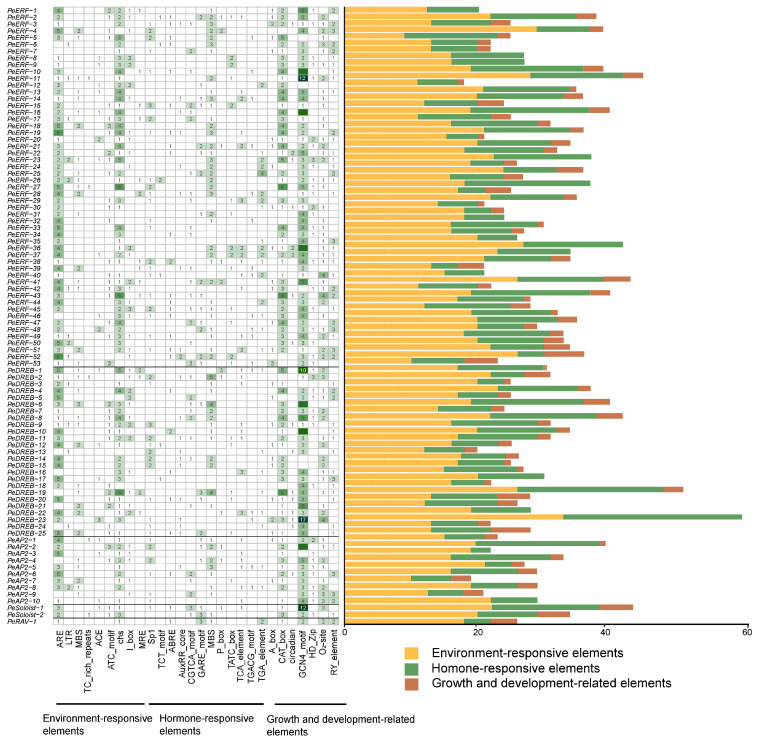
Putative *cis*-elements enriched in the promoter regions of *PeAP2/ERF* family genes. Number of *cis*-acting elements in the promoter regions of individual *PeAP2/ERF* genes. The colors in the left figure indicate the numbers of *cis*-elements. Darker green shades correspond to higher numbers.

Transcription factor-binding sites and regulatory *cis*-elements in the promoter region are pivotal for regulating gene expression. We analyzed the putative regulatory *cis*-elements in the promoters of *PeAP2/ERF* genes using the PlantCARE database 5.0. In addition to the common and core *cis*-elements, such as CAAT and TATA boxes, several *cis*-acting elements related to environmental responses, hormones, and growth and development were identified in the promoters of *PeAP2/ERF* genes (Figure 6). Most of the *PeAP2/ERF* genes showed *cis*-elements associated with environment responses, indicating that *PeAP2/ERF* genes may play a vital role in responding to various abiotic stresses (Figure 6). Moreover, at least five phytohormone responsive *cis*-elements were observed in the promoter of each *PeAP2/ERF* gene, suggesting that *PeAP2/ERF* genes are involved in various hormone signaling pathways in passion fruit (Figure 6). In addition, the number of *cis*-elements responsive to environmental stress and hormones far exceeded those related to growth and development.

### 2.6. Analysis of PeAP2/ERFs Expression Pattern in Postharvest Passion Fruit

**Figure 7:**
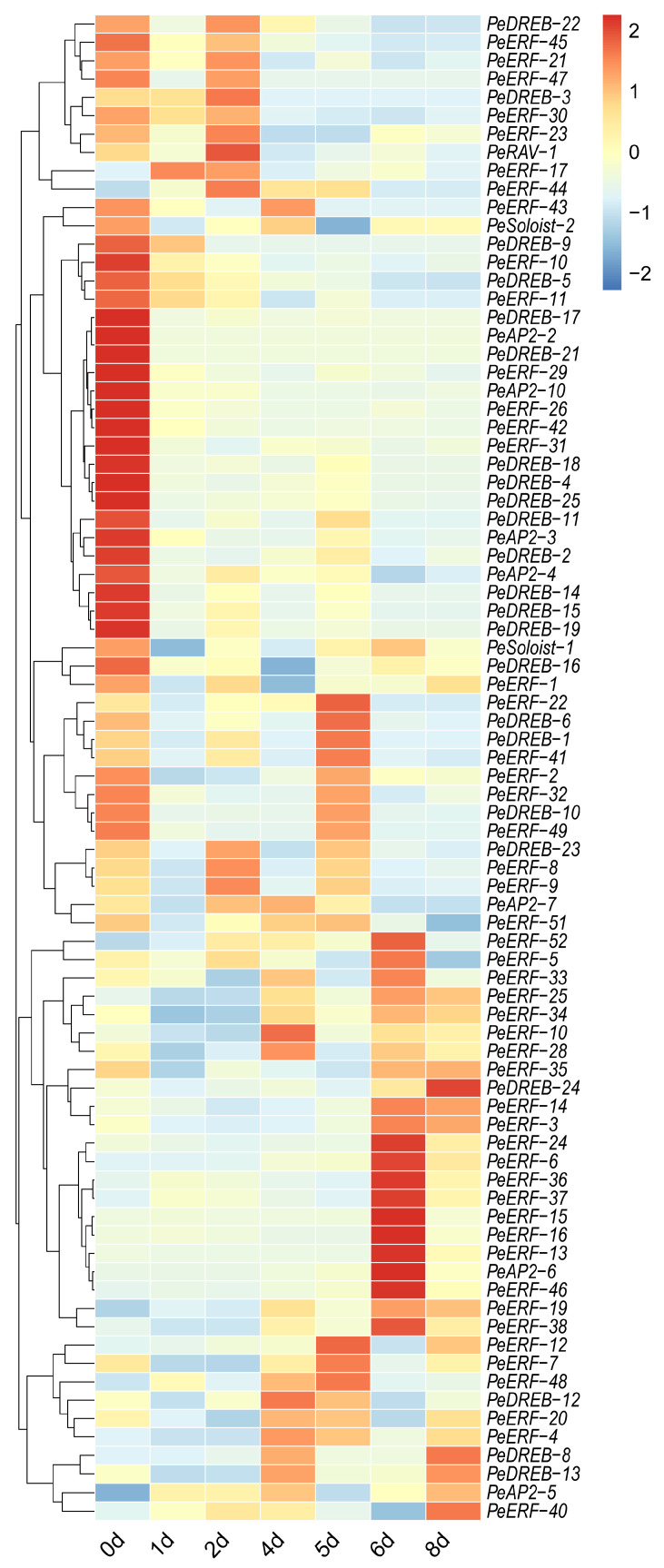
Hierarchical clustering analyses of the *PeAP2/ERFs’* expression in postharvest passion fruits. The relative gene expression levels were determined using the passion fruit housekeeping gene *PeACTIN* as an internal control. The varying expression values of *PeAP2/ERF* genes were analyzed with RNA-seq data. Different colors correspond to expression values.

Previously, we performed a transcriptome analysis in postharvest passion fruit under various storage conditions [22]. To further explore the potential roles of *PeAP2/ERFs* during postharvest storage, we analyzed the expression patterns. *PeAP2-1*, *PeAP2-8*, *PeAP2-9*, *PeDREB-7*, *PeDREB-20*, *PeERF-27*, *PeERF-39*, *PeERF-50,* and *PeERF-53* were not expressed in postharvest fruit. Specifically, 6 *ERF* genes, 12 *DREB* genes, 4 *AP2* genes, and 1 *Soloist* gene were down-regulated during postharvest. The remaining 59 family genes would be up-regulated at least at one time point (Figure 7). However, each individual gene exhibited a distinct expression pattern. For instance, *PeERF-17* peaked in expression at 1 day postharvest (dph); *PeDREB-3*, *PeDREB-23*, *PeRAV-1*, *PeERF-8*, *PeERF-9*, and *PeERF-44* reached their dominant expression levels at 2 dph; *PeAP2-7*, *PeDREB-12*, *PeERF-4*, *PeERF-10*, *PeERF-28* were mainly expressed at 4 dph; at 5 dph, *PeDREB-1*, *PeDREB-6*, *PeERF-7*, *PeERF-12*, *PeERF-22*, *PeERF-41*, *PeERF-48* showed the highest expression; on the 6th day, the expression of *PeAP2-6*, *PeERF-13*, *PeERF-15*, *PeERF-16*, *PeERF-5*, *PeERF-6*, *PeERF-24*, *PeERF-33*, *PeERF-36*, *PeERF-37*, *PeERF-38*, *PeERF-46*, *PeERF-52* had the highest expression; *PeDREB*-*24* and *PeERF-40* attained the highest expression at 8 dph (Figure 7 and Figure S2).

### 2.7. PeAP2-10 Directly Regulates PeSTP6

**Figure 8:**
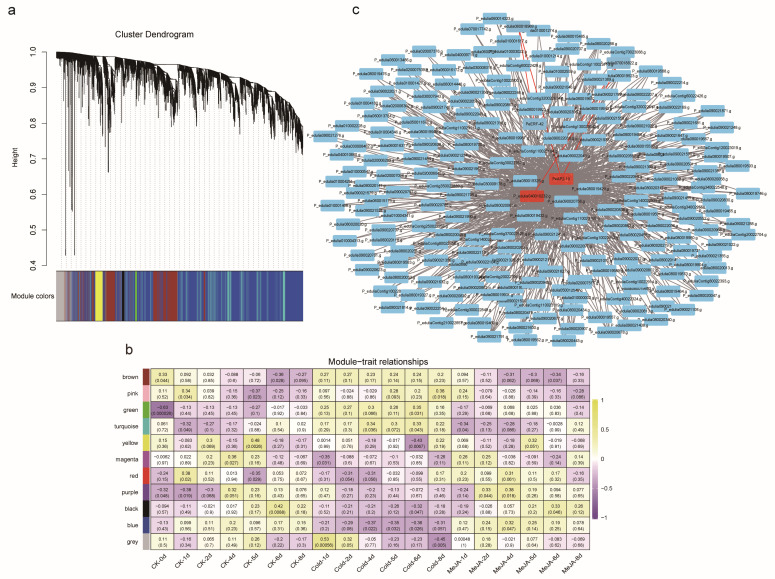
Co-expression analysis. (**a**) Co-expression clustering dendrogram based on expression data generated by a synthetic Gene Regulatory Network (GRN) after 1000 times (iterations). (**b**) Association heatmap between modules and phenotypic traits (cold and MeJA). Each cell contains the correlation coefficient and *p*-value. (**c**) The co-expression network of 218 genes in brown module. The red labeled genes were *PeAP2-10* and *P_edulia040010232.g* (*PeSTP6*), respectively.

To explore the regulation network of *PeAP2/ERF* family genes, we performed co-expression analysis using RNA-seq data described in our previous work. Based on the expression patterns, the genes were divided into 11 modules. Specifically, we focused on PeAP2-10, the only member containing two motif 5 sequences. The expression of *PeAP2-10* was tightly correlated with several genes, including *PeSTP6* (*P_edulia040010232.g*), a sugar transporter-encoding gene homologous to *SlSTP1* (Figure 8 and Figures S2–S4).

**Figure 9:**
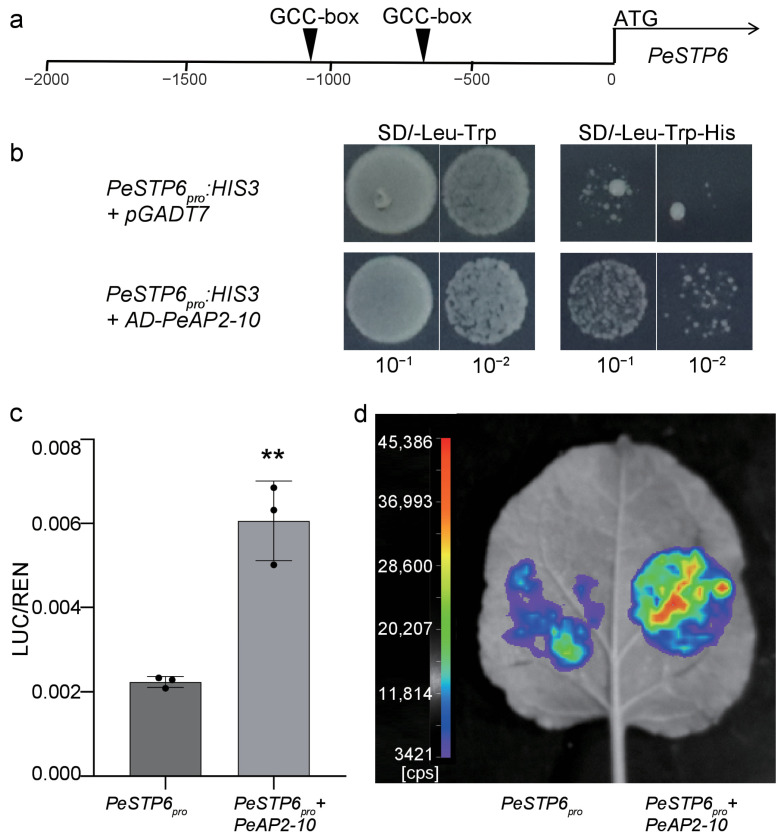
*PeAP2-10* regulates *PeSTP6*. (**a**) The position of the GCC_box identified through *PeSTP6* promoter analysis. (**b**) Yeast one-hybrid (Y1H) assays demonstrated that PeAP2-10 binds to the *PeSTP6* promoters. Dual-luciferase (Dual-LUC) assays (**c**) and imaging (**d**) confirmed that *PeAP2-10* activated *PeSTP6* transcription. In these experiments, *PeSTP6_pro_* and *PeAP2-10* represent the reporter and effector constructs, respectively. Values represent means and standard deviations of three biological replicates, with three technical replicates for each biological replicate. Double asterisks (**) indicate *p* values < 0.01.

The promoter of *PeSTP6* contains two GCC_box *cis*-acting elements (Figure 9a and Figure S5), suggesting that *PeSTP6* serves as a candidate target of AP2 proteins. Subsequently, we performed yeast one-hybrid assays and found that PeAP2-10 bound to the promoter of *PeSTP6* (Figure 9b). A dual luciferase assay confirmed that *PeAP2-10* induced the promoter activity of *PeSTP6* (Figure 9c,d). These data indicate that *PeAP2-10* regulates the expression of *PeSTP6*.

## 3. Discussion

The biological and molecular functions of *AP2/ERF* genes in passion fruit remain unexplored. To elucidate the potential roles of *PeAP2/ERF* genes in passion fruit, we identified 91 *PeAP2/ERF* genes distributed across all 9 chromosomes of its genome (Table 1 and Figure 2). Consistent with findings in Arabidopsis [24], cucumber [25], barley (*Hordeum vulgare*) [26], and tomato [27], PeAP2/ERFs in passion fruit were classified into five subfamilies based on their phylogenetic relationships (Figure 1). Most AP2/ERF members were found in the AP2, ERF, and DREB subfamilies. This distribution aligns with observations in desert legumes (153 members: with 24 AP2, 59 DREB, and 68 ERF) [28]. *Rhododendron* has a total of 120 AP2/ERF family members, including 17 AP2s, 44 DREBs, and 53 ERFs, which constitute approximately 95% of the total number of AP2/ERF members [29]. This indicates that the AP2, ERF, and DREB subfamilies account for a large proportion of the total AP2/ERF family members and may play a broad role.

Tandem and segmental duplication events, which arise from whole genome duplication (WGD) through polyploidization or local chromosomal rearrangements, contribute to gene family expansion and evolution [30]. The maximum number of collinear orthologs was identified between passion fruit and Arabidopsis, indicating that *PeAP2/ERF* genes are conserved and probably share common ancestors (Figure 3). Duplication events likely occurred in a common ancestor, and duplicated genes tend to exhibit a closer relationship. Our phylogenetic tree revealed that 10 out of the 12 gene pairs in tandem duplication events were derived from the ERF subfamily (Figure 2).

The number of *PeAP2/ERF* genes in passion fruit exceeds that in *Brassica napus* (87 members) [31] and *Penniseagraria* (78 members) [32], yet it is less than that in tomato (134 members) [27], rice (170 members) [33], and wheat (322 members) [34]. This variation highlights the diversity in the distribution of *PeAP2*/*ERF* genes among different species. It is estimated that passion fruit diverged from the *Euphorbiaceae* lineage approximately 65 million years ago (MYA) and from *Salicaceae* approximately 60 MYA [35]. Moreover, genomic analysis has demonstrated that passion fruit experienced a recent whole-genome duplication (WGD) event after diverging from both *Euphorbiaceae* and *Salicyliaceae* [36,37]. The relatively small number of *AP2*/*ERF* genes implies the occurrence of gene loss events, which often follow WGD, during the evolutionary process of passion fruit. Similar assumptions have been made for *Pennisetum glaucum*, which naturally has a strong tolerance to various environmental stresses but contains a small number of *AP2*/*ERF* superfamily members [32]. However, the specific mechanisms by which different *AP2*/*ERF* family members contribute to the domestication and adaptation of passion fruit remain to be clarified.

Passion fruit is cultivated in tropical and subtropical areas, and its fruits are harvested at the early age [21,35]. Therefore, understanding the fruit characteristics during the postharvest maturation period is essential. Previous studies have reported that *AP2*/*ERF* genes play crucial roles in the preservation of postharvest fruits. For instance, the expression of *ERFs* is important in the ripening and senescence of peach during postharvest storage [38]. In red-fruit loquat, *EjERF39* responds to environmental conditions, which is involved in the lignification of immature loquat fruits [39]. The specific expression of *AP2/ERF* genes had been noticed during the rapid softening of postharvest papaya pulp [40]. Recently, we discovered that the quality and flavor of postharvest passion fruit were significantly enhanced following low-temperature and MeJA treatment [22]. In this study, we characterized the induced expression of *PeAP2-10* and identified the *PeAP2-10-PeSTP6* expression cascade. However, the specific functions of *AP2/ERF* genes in this process remain unclear.

Postharvest preservation is essential for fruit quality and flavor. In this study, most members of the *PeAP2/ERF* family responded to postharvest storage, exhibiting varying degrees of response, except for *PeAP2-1*, *PeAP2-8*, *PeAP2-9*, *PeDREB-7*, *PeDREB-20*, *PeERF-27*, *PeERF-39*, *PeERF-50,* and *PeERF-53* (Figure 7). More than 60% (59 out of 91) of family members were up-regulated with the extension of postharvest time (Figure 7). Notably, *PeAP2-10* expression correlated with *PeSTP6* (Figure S2), a putative ortholog of the tomato sucrose transporter gene governing soluble solid content [41]. Transactivation assays confirmed *PeAP2-10*’s role to induce *PeSTP6* (Figure 9), implicating it in sugar transport and flavor modulation. These findings highlight *PeAP2/ERFs* as potential regulators of postharvest metabolic dynamics in passion fruit.

## 4. Materials and Methods

### 4.1. Plant Materials

Mature fruits of passion fruit (*Passiflora edulis* Sims) cultivar Tainong 1 were collected from the Passion Fruit Science and Technology Backyard in Baisha, Hainan, China. The fruits were stored for 0, 1, 2, 4, 5, 6, or 8 days. Three biological replicates were utilized, with each consisting of six fruits [22].

### 4.2. Identification of the PeAP2/ERF Family Members in Passion Fruit

The genome information of passion fruit was obtained from a previous study [21]. The amino acid sequences of 141 AtAP2/ERF proteins in the *A. thaliana* were downloaded from the TAIR database (https://www.arabidopsis.org/, accessed on 28 July 2023), and the sequences of 134 SlAP2/ERF proteins in the *Solanum lycopersicum* were retrieved from the Sol Genomics Network database (https://solgenomics.net/, accessed on 28 July 2023). These sequences were subsequently employed as queries for a BLAST search against the passion fruit genome, utilizing an E-value threshold of <e^−10^ and a sequence identity of >50% [42]. Meanwhile, Hidden Markov Model (HMM) (version 3.0, http://hmmer.janelia.org/) was used with the default parameters for the identification of AP2/ERF proteins. The AP2 domain (PF00847) was retrieved from the Pfam database (http://pfam.xfam.org, accessed on 30 July 2023). The presence of the AP2 domain in each protein was confirmed using the SMART (http://smart.embl-heidelberg.de/, accessed on 30 July 2023).

### 4.3. Phylogenetic Analysis

Phylogenetic analysis was conducted using amino acid sequences of the identified PeAP2/ERFs and AtAP2/ERFs from *A. thaliana*. A phylogenetic tree was constructed using the Neighbor-Joining (NJ) method as implemented in MEGA12 software, with a bootstrap of 1000 replicates. Subsequently, the tree was subsequently visualized using iTol software v7 (https://itol.embl.de/, accessed on 30 July 2023) [43].

### 4.4. Chromosomal Distribution and Collinearity Analysis

The chromosomal locations of *PeAP2/ERF* genes were visualized using MapChart (version 2.32) [44]. MCScanX (v1.0.0) analysis [45] was employed to identify gene families containing *PeAP2/ERF* copies, and CIRCOS [46] was utilized to construct the collinearity map of *PeAP2/ERF* genes.

### 4.5. Gene Structure and Conserved Motifs of PeAP2/ERF

The gene structures of *PeAP2/ERF* were visually mapped based on the passion fruit genome annotation using the GSDS online tool (https://gsds.gao-lab.org/, accessed on 30 July 2023) [47]. Additionally, the protein sequences of PeAP2/ERF were submitted to TBtools [48] for motif prediction, with the motif discovery number set to 10 and all other parameters set to default.

### 4.6. Prediction of Cis-Elements in PeAP2/ERF Genes Promoter

The 2000-bp upstream of the start codon of the *PeAP2/ERF* family genes were submitted to PlantCARE (http://bioinformatics.psb.ugent.be/webtools/plantcare/html/, accessed on 4 August 2023) for the prediction of *cis*-acting elements. Subsequently, the results from PlantCARE were subsequently sorted and simplified [49].

### 4.7. Yeast One-Hybrid

The promoter of the *PeSTP6* gene was recombined into the pHIS2 plasmid to generate the *PeSTP6_pro_:HIS3* construct. The CDS sequence of *PeAP2-10* was inserted into the pGADT7 to generate the *AD:PeAP2-10* construct. The recombinant plasmids were co-transformed into yeast strain Y187. Positive single colonies were selected. After identification, the SD/-Leu-Trp and SD/-Leu-Trp-His concentration determined by screening was used for dot-plate and interaction verification. The cells were incubated at 30 °C for 3–4 d and then photographed and recorded.

### 4.8. Transient Dual Luciferase Reporter Assays

The promoter of *PeSTP6* was cloned into the PJG077 vector [22], and the CDS of *PeAP2-10* was inserted into the pEAQ-HT-DEST2 vector to generate the reporter and the effector constructs, respectively. Then, these constructs were co-expressed in the tobacco leaves. After incubation for 44 h, the transfected cells were collected and homogenized in 300 μL of passive lysis buffer. A 10 μL aliquot of the crude extract was mixed with 50 μL of luciferase assay buffer, and the Firefly Luciferase (LUC) activity was measured using a Varioskan LUX (Thermo Scientific, Waltham, MA, USA). Stop and Glow Buffer (50 μL) was subsequently added to the reaction solution and the Renilla Luciferase (REN) activity was measured. The LUC/REN ratio was calculated to represent the relative activity of the transcription factors [22].

### 4.9. RNA Extraction and qRT-PCR

Total RNA in each sample was extracted, using the TRIzol reagent kit (Vazyme, Nanjing, China). All RNA was purified, using the Hifair^®^ II 1st Strand cDNA Synthesis Kit (gDNA digester plus) (Yeasen, Shanghai, China) for first-strand cDNA synthesis. Quantitative RT-PCR was performed using the 2×SYBR Green qPCR Mix (SparkJade, Jinan, China). The Quantstudio™ 7 Flex Real-time PCR system (Applied Biosystems, Carlsbad, CA, USA) was used for detection. Samples were normalized using the *PeActin* gene (*LOC8268098/XP_002531173.1*) as an endogenous control. qRT-PCR primers were designed using Primer 3 based on the CDS sequences of the *PeAP2/ERF* genes (Table S1). Three biological replicates were analyzed, each containing six technical replicates. The 2^−ΔCt^ comparative method was used to calculate the relative transcript levels in the samples [50,51].

### 4.10. Co-Expression Analysis

Gene co-expression network analysis was performed using pairwise correlations between quantile-normalized expression profiles derived from published RNA-seq data [22]. Scale-free weighted gene co-expression networks were constructed using the “WGCNA” package. The WGCNA analysis was conducted with the WGC-NAshiny plugin in TBtools, with the following parameters: RNA-seq data from passion fruit peel treated at 26 °C, 8 °C, and 100 mM MeJA for 0, 1, 2, 4, 5, 6, and 8 days; sample percentage = 0.9; expression cutoff = 1; filter method = MAD (Median Absolute Deviation); reserved genes = 20,000; final power selection = 14; minimum module size = 30; and module cuttree height = 0.4. The analysis was performed using the RNA-seq data to generate the final results.

Pearson’s correlations were employed to avoid assuming linear relationships between co-expressed genes, and genes displaying standard deviation equal to zero were eliminated in both empirical and simulated datasets. Unsupervised hierarchical clustering was applied to detect modules of highly co-expressed genes following the method described [52]. Association heatmaps and co-expression network analyses were performed using TBtools software (v2.121) and RNA-seq data.

### 4.11. Statistical Analysis

All results were presented as means ± standard deviation (SD) of at least three biological replicates. Student’s *t*-test was applied to analyze the significant differences (*, *p* < 0.05; **, *p* < 0.01).

## 5. Conclusions

In conclusion, this study provides a comprehensive characterization of the *PeAP2/ERF* gene family in passion fruit, revealing its significant role in regulating postharvest processes and flavor enhancement. The identification of 91 *PeAP2/ERF* genes and their classification into distinct subfamilies underscores the evolutionary conservation and functional diversity of this transcription factor family. Our findings indicate that *PeAP2/ERF* genes, particularly *PeAP2-10*, are responsive to postharvest conditions and may play a pivotal role in sugar transport, thereby influencing the flavor profile of passion fruit. The insights gained from this research not only enhance our understanding of the molecular mechanisms underlying fruit quality but also provide a foundation for future studies aimed at improving postharvest management practices. Ultimately, elucidating the specific functions of *PeAP2/ERF* genes will be essential for developing strategies to optimize the quality and marketability of passion fruit, contributing to the sustainability of this important crop in tropical and subtropical regions.

## Figures and Tables

**Table 1:** Identification and characterization of *PeAP2/ERF* genes and their predicted proteins in passion fruit.

Gene ID	ID	Chr No.	Protein Length (aa)	CDS (bp)	MW (Da)	*p*I	GRAVY
*P_edulia010004144.g*	*PeAP2-1*	Chr1	678	2037	75,028	6.84	−0.75
*P_edulia020006708.g*	*PeAP2-2*	Chr2	589	1770	63,971	7.8	−0.39
*P_edulia030008406.g*	*PeAP2-3*	Chr3	510	1533	56,401	6.25	−0.61
*P_edulia030008511.g*	*PeAP2-4*	Chr3	417	1254	46,541	9.72	−0.82
*P_edulia040010127.g*	*PeAP2-5*	Chr4	546	1641	60,563	7.14	−0.74
*P_edulia050011325.g*	*PeAP2-6*	Chr5	351	1056	39,533	5.16	−0.66
*P_edulia060015442.g*	*PeAP2-7*	Chr6	682	2049	74,340	7.56	−0.62
*P_edulia060015844.g*	*PeAP2-8*	Chr6	721	2166	78,270	6.54	−0.71
*P_edulia070018496.g*	*PeAP2-9*	Chr7	310	933	34,092	6.18	−0.66
*P_edulia080019291.g*	*PeAP2-10*	Chr8	411	1236	46,358	8.36	−0.68
*P_edulia010000572.g*	*PeDREB-1*	Chr1	236	711	25,967	5.01	−0.42
*P_edulia010001596.g*	*PeDREB-2*	Chr1	1209	3630	133,140	10.39	−0.24
*P_edulia010003920.g*	*PeDREB-3*	Chr1	230	693	25,106	4.82	−0.64
*P_edulia040009646.g*	*PeDREB-* *4*	Chr4	240	723	26,964	6.51	−0.55
*P_edulia040010723.g*	*PeDREB-* *5*	Chr4	310	933	33,942	8.35	−0.45
*P_edulia040010839.g*	*PeDREB-* *6*	Chr4	234	705	26,077	5.22	−0.75
*P_edulia040011090.g*	*PeDREB-* *7*	Chr4	190	573	20,815	9.71	−0.69
*P_edulia050011280.g*	*PeDREB-* *8*	Chr5	286	861	31,335	5.22	−0.71
*P_edulia050011897.g*	*PeDREB-* *9*	Chr5	218	657	24,004	6.73	−0.77
*P_edulia050012013.g*	*PeDREB-1* *0*	Chr5	241	726	27,470	4.87	−0.87
*P_edulia050012014.g*	*PeDREB-1* *1*	Chr5	243	732	26,281	4.67	−0.54
*P_edulia050012164.g*	*PeDREB-1* *2*	Chr5	297	894	33,242	8.44	−0.57
*P_edulia060013532.g*	*PeDREB-1* *3*	Chr6	439	1320	48,352	8.76	−0.60
*P_edulia060014565.g*	*PeDREB-1* *4*	Chr6	215	648	23,342	4.66	−0.43
*P_edulia060014877.g*	*PeDREB-1* *5*	Chr6	215	648	23,376	4.56	−0.48
*P_edulia060015588.g*	*PeDREB-* *16*	Chr6	292	879	32,099	5.74	−0.41
*P_edulia070016972.g*	*PeDREB-* *17*	Chr7	194	585	20,703	4.84	−0.39
*P_edulia070017934.g*	*PeDREB-* *18*	Chr7	287	864	31,127	4.48	−0.71
*P_edulia070018079.g*	*PeDREB-* *19*	Chr7	215	648	23,856	4.26	−0.70
*P_edulia070018189.g*	*PeDREB-2* *0*	Chr7	251	756	27,266	4.74	−0.55
*P_edulia070018581.g*	*PeDREB-2* *1*	Chr7	244	735	26,542	4.99	−0.47
*P_edulia090020954.g*	*PeDREB-2* *2*	Chr9	262	789	28,430	5.14	−0.55
*P_edulia090021123.g*	*PeDREB-2* *3*	Chr9	208	627	22,834	4.27	−0.46
*P_eduliaContig200022650.g*	*PeDREB-2* *4*	Contig	440	693	48,313	6.84	−0.59
*P_eduliaContig30022789.g*	*PeDREB-2* *5*	Contig	256	1123	33,592	9.83	−0.55
*P_edulia010000150.g*	*PeERF-1*	Chr1	251	1176	27,267	4.65	−0.44
*P_edulia010001731.g*	*PeERF-2*	Chr1	391	1107	43,201	5.54	−0.89
*P_edulia010001854.g*	*PeERF-3*	Chr1	368	966	41,985	4.73	−0.59
*P_edulia010001961.g*	*PeERF-4*	Chr1	321	1248	35,875	4.99	−0.45
*P_edulia010002055.g*	*PeERF-5*	Chr1	415	732	46,607	6.54	−0.64
*P_edulia010002171.g*	*PeERF-6*	Chr1	243	597	26,686	6.25	−0.93
*P_edulia010003619.g*	*PeERF-7*	Chr1	127	900	14,151	9.72	−0.85
*P_edulia010004885.g*	*PeERF-8*	Chr1	299	651	33,729	7.14	−0.75
*P_edulia010005010.g*	*PeERF-9*	Chr1	216	651	23,667	7.33	−0.75
*P_edulia010005188.g*	*PeERF-10*	Chr1	249	750	28,127	4.79	−0.76
*P_edulia010005329.g*	*PeERF-11*	Chr1	216	645	23,653	8.07	−0.84
*P_edulia020006460.g*	*PeERF-12*	Chr2	382	423	42,764	5.06	−0.58
*P_edulia030007663.g*	*PeERF-13*	Chr3	140	747	15,549	10.28	−0.72
*P_edulia030007664.g*	*Pe* *ERF-14*	Chr3	257	774	28,824	6.51	−0.78
*P_edulia030007668.g*	*Pe* *ERF-15*	Chr3	248	639	27,691	9.12	−0.71
*P_edulia030007669.g*	*Pe* *ERF-16*	Chr3	212	618	23,722	4.68	−0.68
*P_edulia030009441.g*	*PeERF-1* *7*	Chr3	205	678	23,055	5.95	−0.59
*P_edulia040009825.g*	*PeERF-1* *8*	Chr4	225	945	24,644	7.09	−0.82
*P_edulia040010539.g*	*PeERF-1* *9*	Chr4	314	978	35,157	5.89	−0.72
*P_edulia040010561.g*	*PeERF-* *20*	Chr4	325	543	36,419	6.68	−0.86
*P_edulia040010637.g*	*PeERF-* *21*	Chr4	274	825	30,768	6.79	−0.56
*P_edulia040010735.g*	*PeERF-* *22*	Chr4	180	957	20,655	7.25	−0.53
*P_edulia040011072.g*	*PeERF-* *23*	Chr4	318	792	35,009	5	−0.71
*P_edulia040011079.g*	*PeERF-2* *4*	Chr4	267	804	29,686	7.95	−0.31
*P_edulia050011662.g*	*PeERF-2* *5*	Chr4	263	438	28,058	8.62	−0.96
*P_edulia050011732.g*	*PeERF-2* *6*	Chr5	145	558	16,267	7.51	−0.77
*P_edulia050011872.g*	*PeERF-2* *7*	Chr5	275	597	30,447	8.6	−0.50
*P_edulia050011874.g*	*PeERF-2* *8*	Chr5	185	828	20,952	8.03	−0.63
*P_edulia050012238.g*	*PeERF-2* *9*	Chr5	198	897	21,956	9.5	−0.54
*P_edulia050012239.g*	*PeERF-* *30*	Chr5	337	1014	37,775	5.96	−0.60
*P_edulia050012240.g*	*PeERF-* *31*	Chr5	298	765	32,899	8.61	−0.43
*P_edulia060013160.g*	*PeERF-* *32*	Chr5	254	1203	27,510	9.13	−0.82
*P_edulia060013663.g*	*PeERF-* *33*	Chr6	400	1203	44,660	4.45	−0.64
*P_edulia060013843.g*	*PeERF-3* *4*	Chr6	400	1071	43,717	8.58	−0.63
*P_edulia060014975.g*	*PeERF-3* *5*	Chr6	356	552	38,670	8.07	−0.37
*P_edulia060015066.g*	*PeERF-36*	Chr6	183	1299	20,099	10.63	−0.69
*P_edulia060015079.g*	*PeERF-3* *7*	Chr6	432	1026	47,198	8.45	−0.78
*P_edulia060015213.g*	*PeERF-3* *8*	Chr6	234	705	26,272	5.24	−0.65
*P_edulia060015996.g*	*PeERF-3* *9*	Chr6	342	570	36,653	6.31	−0.68
*P_edulia070017176.g*	*PeERF-* *40*	Chr7	189	615	21,274	10.54	−0.73
*P_edulia070017525.g*	*PeERF-* *41*	Chr7	204	726	22,966	7.66	−0.54
*P_edulia070017902.g*	*PeERF-* *42*	Chr7	190	573	21,462	10.11	−0.77
*P_edulia070018289.g*	*PeERF-* *43*	Chr7	187	564	20,782	9.37	−0.62
*P_edulia070018452.g*	*PeERF-4* *4*	Chr7	241	1161	25,808	6.54	−0.68
*P_edulia080019076.g*	*PeERF-4* *5*	Chr8	386	504	42,800	4.76	−0.70
*P_edulia080019171.g*	*PeERF-4* *6*	Chr8	167	441	18,581	7.32	−0.68
*P_edulia090020522.g*	*PeERF-4* *7*	Chr9	118	357	13,273	PI	−0.66
*P_edulia090020764.g*	*PeERF-4* *8*	Chr9	393	1182	16,176	4.62	−0.63
*P_edulia090020847.g*	*PeERF-4* *9*	Chr9	194	585	43,325	7.56	−0.63
*P_edulia090021477.g*	*PeERF-* *50*	Chr9	205	618	22,911	6.18	−0.74
*P_edulia090022050.g*	*PeERF-* *51*	Chr9	321	966	22,228	8.36	−0.42
*P_eduliaContig100022618.g*	*PeERF-* *52*	Contig	230	543	34,318	7.38	−0.78
*P_eduliaContig240022563.g*	*PeERF-* *53*	Contig	180	1188	25,530	7.8	−0.49
*P_edulia020006614.g*	*PeRAV-1*	Chr2	361	1086	41,452	5.16	−0.64
*P_edulia010005093.g*	*PeSoloist-1*	Chr1	231	696	25,920	4.67	−0.78
*P_edulia060014445.g*	*PeSoloist-2*	Chr6	229	690	25,703	9.13	−0.88

## Data Availability

Data are contained within the article and Supplementary Material.

## Supplementary Materials

The following supporting information can be downloaded at: https://www.mdpi.com/article/10.3390/plants14050645/s1. Figure S1: The conserved motifs of amino acid sequences identified by MEME 5.5.7 software; Figure S2: qRT-PCR validation of the differentially expressed genes and the relative expression level of *PeAP2-10* and *PeSTP6*; Figure S3: Phylogenetic relationships of STP proteins from Arabidopsis and passion fruit; Figure S4: Amino acid alignment analysis of SlSTP1 and PeSTP6; Figure S5. Promoter sequence of *PeSTP6*. Table S1: Primers sequences used in this study.
