# Supplementary material for: Genome-Wide Identification and Functional Analysis of AP2/ERF Gene Family in Passiflora edulis Sims
Source: Plants (Basel). 2025 Feb 20;14(5):645. doi: 10.3390/plants14050645 (PMC11901831; doi:10.3390/plants14050645)
Supplement: Supplementary file 1 [file plants-14-00645-s001.zip › plants-3428233-supplementary.pdf]

Table S1. Primers sequences used in this study.

Primer name	Forward Primer	Reverse Primer
PeERF-10	TGCTCCAAGTAATCGGCTCC	TATGAAGATGGGTGCTCGCC
PeERF-19	AGCACAGAGGAAGGAACCAA	GACAAGCCATCTCCTACGCA
PeERF-48	CTTGGGTCACGTATCTCCGC	GCTAAACCTCTCGCTTTCGC
PeERF-23	CTTCGTCCCAGAACCCCTTC	GGTCCGTGATCCTTGAGAGC
PeAP2-10	ACTGCCTACGATATGGCTGC	AGGATTGAAGCAGAAGGCCC
PeSTP6	CTTCGCTTGCTTTGGCCTTT	AGCGTGCTCGAAGAAATGGA
PeActin	AGGTGGGTAACAGGATTATC	TGGCTGTCTTTTGGTGCTG
PeAP2-10	agGGTCTCAattcGATGTCTCATCAACCGCCAC	agGGTCTCAgctcCGCTCTGCTCTAGCTGCTG
PeSTP6	agGGTCTCAattcACGGGACAGCTTCAAAAGTG	agGGTCTCAgctcGATGGCCGCAAGTGAATGTC

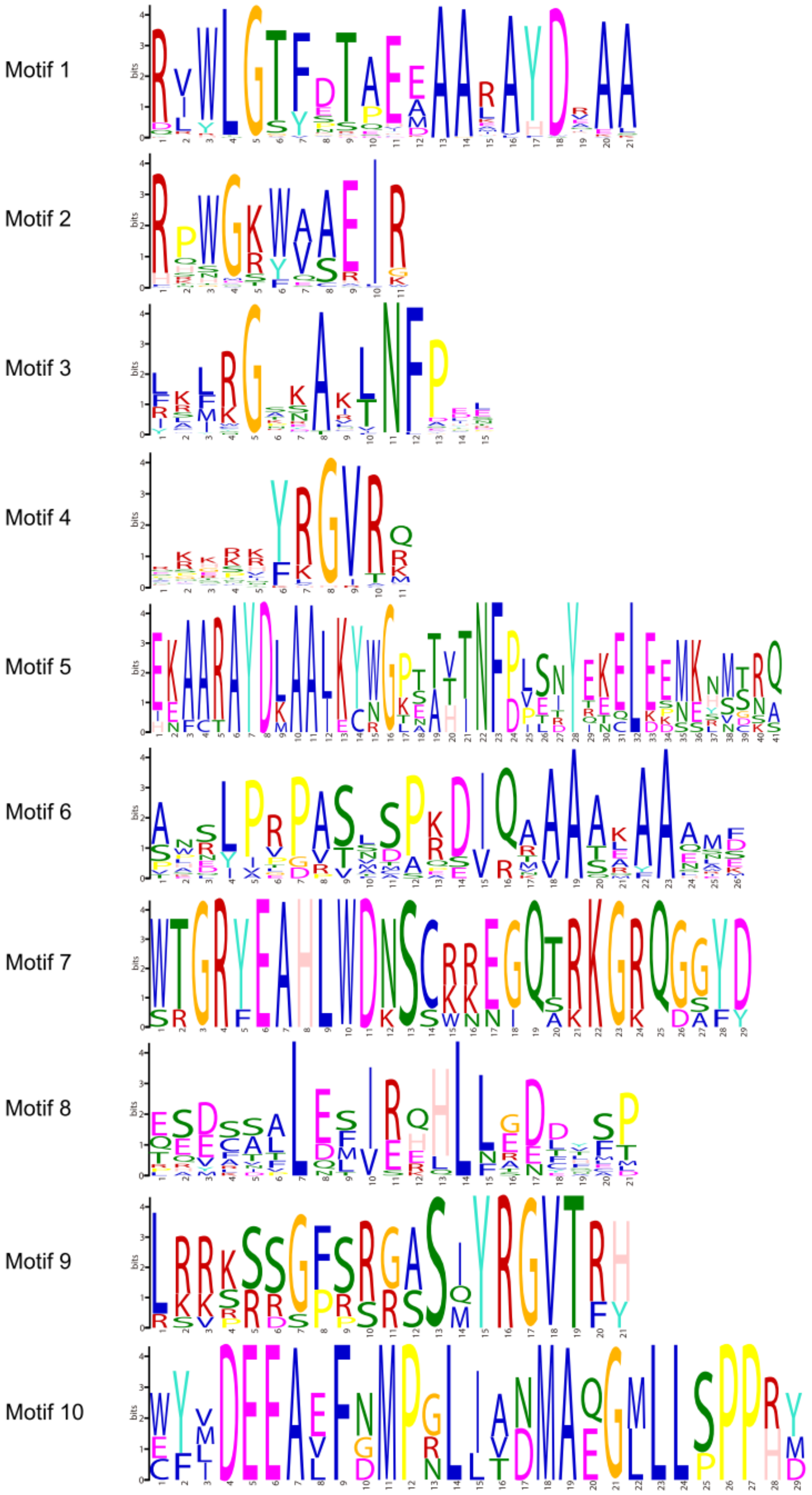

Figure S1. The conserved motifs of amino acid sequences identified by MEME software. Motifs 1-10 correspond to different amino acid secondary motif sequences, respectively. The height of each letter in the figure, measured in bits, is proportional to the frequency of occurrence of the corresponding letter at the position. The letters in each position are arranged in order of conservation from large to small.

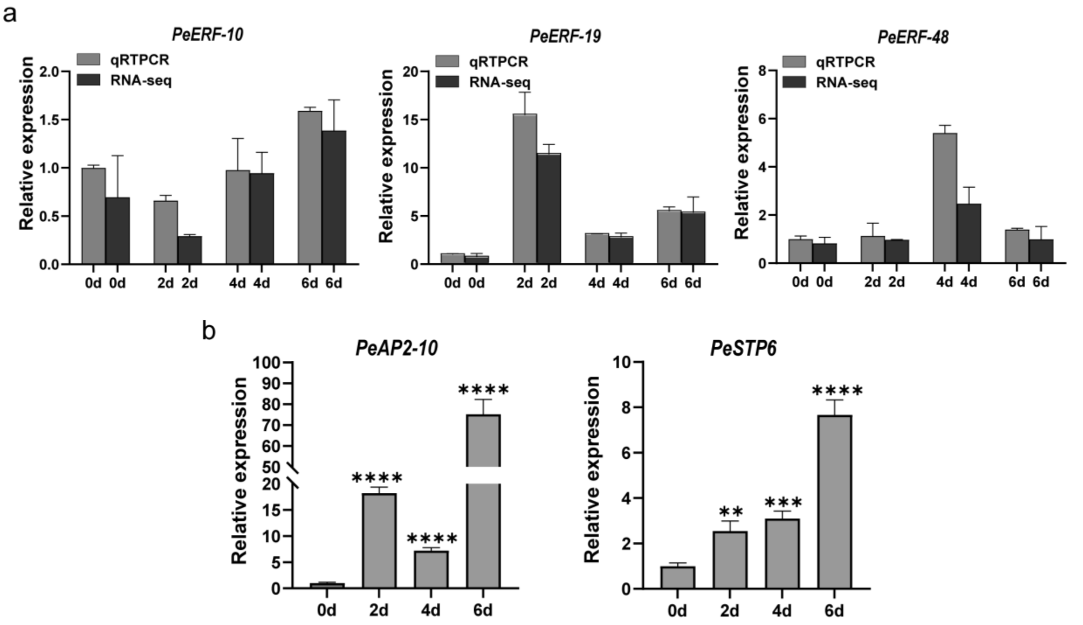

Figure S2. qRT-PCR validation of the differentially expressed genes and the relative expression level of *PeAP2-10* and *PeSTP6*.

(a) Three *PeAP2/ERF* family genes, *PeERF-10*, *PeERF-19*, and *PeERF-48* were randomly selected to compare the results of two different measurement methods, qRTPCR (gray) and RNA-seq (black), under the same conditions. (b) *PeAP2-10* and *PeSTP6* are expressed in passion fruit over time. Values represent means and standard deviations of three biological replicates, with three technical replicates for each biological replicate. Student's *t*-test was applied to analyze the significant differences (*, $P < 0.05$; **, $P < 0.01$; ***, $P < 0.001$; ****, $P < 0.0001$).

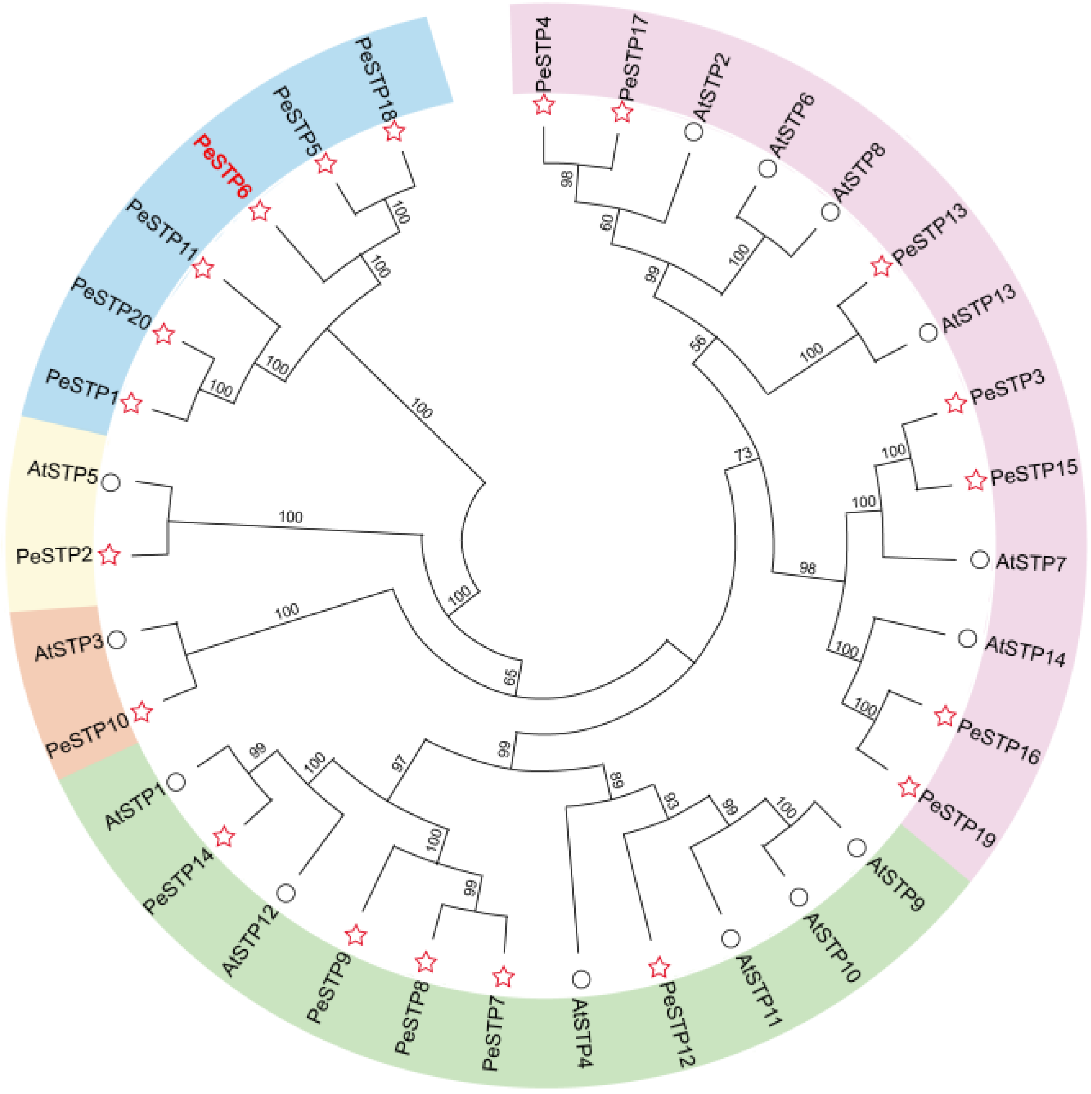

Figure S3. Phylogenetic relationships of STP proteins from Arabidopsis and passion fruit.

A total of 14 AtSTPs (circle ring) from Arabidopsis and 20 PeSTPs (pentagonal ring) from passion fruit were used to construct a phylogenetic tree using the Neighbor-Joining (NJ) method implemented in MEGA12 software. The numbers on the branches represent bootstrap values calculated from 1,000 replicates. PeSTP6 (bold red) is the gene we described here.

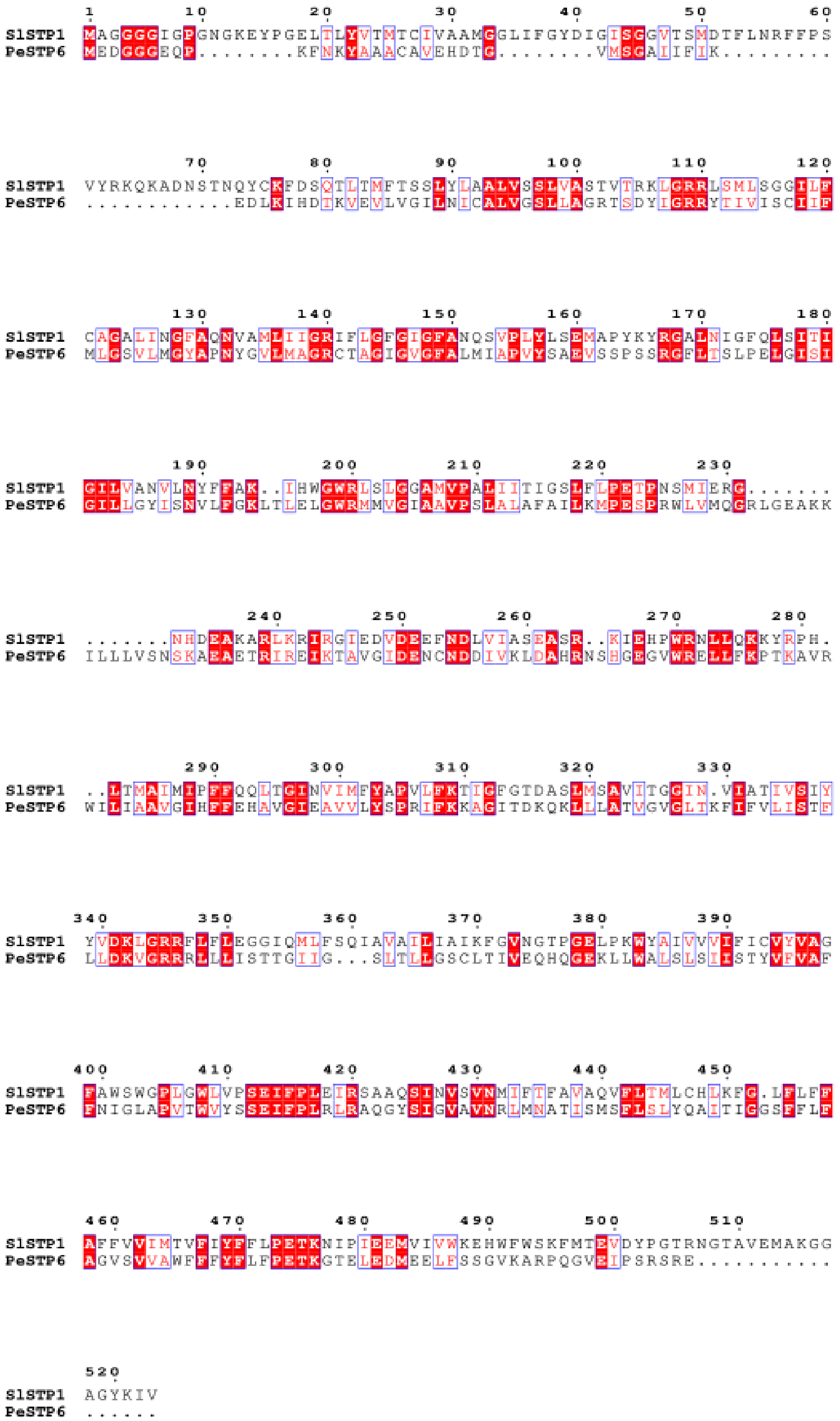

Figure S4. Amino acid alignment analysis of SlSTP1 and PeSTP6.

Numbers in the figure are the amino acid order of SlSTP1. The regions where SlSTP1 and PeSTP6 amino acid sequences are identical are shown in red.

-2000 TAACTTTCAACTCCTTATCCATACGGGACAGCTTCAAAAGTGAAGATATGTTATTTTTCCAGCAATAAATT

-1930 ATCTTCAAATCTAGATTTCTATTTTTGTATTTTATAAAGTAATTGTGTACAACAAGCCACCTAAGTGGCC

-1860 TTCCGCATGCATGCATCAATCCTTCTTGGGTGGACACTTTATTTGTGTGTGTAAGATGGATACTATAATT

-1790 TGTCTGTTTGACACAGATGCATGAATTAATTGAATGATATTTTTAATAAAAAAATGTCTAATATGTCCAA

-1720 CACCATCAAGTCAATATTGATAATCATTTTTAGGAATCATGTTGGTGCAGTAGTTTTGAATATGTTACAA

-1650 ATCCTTAATTTACGATTTGGTTTCAATTCCTATATCACAACGAATGAAATTGATCTAACCAATCAAATCA

-1580 TAATATTCATTTTTTTTGAAAAAAAATCACTCAGGAAAACATTTTAATCAATTAATATATTTTTAATCAA

-1510 TTTTAATACTTTTTCACGGGCACTGTCCAACGTTGCAGTCTATTTCCTTTGCTAGCTCTCAAAATACGTA

-1440 GCACCCAGAAAGGAGAATCTCTACAAAATTTGGTTTCTGTAATAATAAATTATTGTTGCTAAATAGAGAT

-1370 ATTATTCCTTGGCATAAACAAACAGTTAAATAATGTTCTGCCCAGTTTTAGTAGGCATTGATCACGGTTC

-1300 ATATTCTGCCACCTTACTTGCCCATACCTGCCATAATTTTGCGGGTCTTTATCAAAAAGATGTGGGTTTG

-1230 AATGACCCTAAGATTCTCTCCTTGCAACTGACCAATTGCGCGAGACCTTTATTACAACGCTTTCCATCTT

-1160 CTTCTTGGCCTCATTTTCTGGATCCACTATCGCACTCTTGCTTCCAGTTTTGACTTTTGACTCTTCTCCT

-1090 CCAGGATACTGTTTCACCCCCGAGTCTCCGCCACTGCGTGTCGTTTTCTGTCATACTCTATTGTGGTTGA
GCC-box

-1020 TACTTTATAGCTATTGGCCATGCTTTGCACTATCCTTTCTCATGGACAATAGTCAGTAACTGTATTCGAC

-950 GACCTGATATCTATCTCCTTTCTACTCGTAGTCACTCATTTTCTAGGTCGTTGTCAATTTGACTCGACAG

-880 GTATAAGGATATATACATAAACATTACTAAAACTTGTACGGGCTGATGATAATATGTATTTGTTCCTAAA

-810 AATATATTCTATTGGAATCCAAGACTACCAAATTAATACATGAGATAGACGTCTGGCTTGGGCTGATAAC

-740 AGTAAAAAAATGATTGTATTTTCGTAAATATTTAATCATAATTCGGAACATACCGATCAAACCCAAAAAAA

-670 AAAAAAAATTATTTCTAATATAAATATAAAGGCAAAGACTATATATGAGACCGTCCATTCTGGTTGACTA
GCC-box

-600 TGGATAGAGGAGGAGTTTGCCTTGTCAATAATCTTATCCAAAATAAATATACCTTCTGTAGATCAATGGC

-530 CAAGTTCGTACGACCCGTGTAAACGGAAAGTCTGACAAAGATATTATCGCATATACGTGGATTAAGAACA

-460 TCCTAATTGCGAGATTATAGTTCAACGTCACTAAACCTGTTTCGTTCCATTGCCTAAATATAGTGGATAA

-390 GGTTTAGAGAAGGCTTAAAACATTAATTTATTTAATTCTGTTTGAACAAAAAGGAAATGTGGTTTCCACG

-320 ATCAATTAGGAATTTGTTTGATTTTTGTGGTACAGCAAAGCAAAGTGTACGTACAATTGTTTTCAGTTGG

-250 GAAAAGGGTTCACAAGTCCTTGGTTGCATGCTACAAAAGCTATAGGAATGACATTCACTTGCGGCCATCC

-180 TGTTGCTTTTTTGCCTATAAAGGAAACTGAGACGCAAGGCTTTTTGTGCGCATAGGCATATCTGCAGAAA

-110 ACACGCTTACGTATTAGCTATAACGCAGATAGTTAAGTGTTAGCTTCTTTCTTGTTTGTTTCTAGGATCG

-40 AACTCAGAGGTAAGAGAAATCAATACTTGTCTGGATATTA

Figure S5. Promoter sequence of *PeSTP6*. GCC-box are annotated.
